# From fishing village to atomic town and present: A grounded theory study

**DOI:** 10.1371/journal.pone.0310144

**Published:** 2024-11-27

**Authors:** Elfriede Derrer-Merk, Lakshay Jain, Omid Noori-Kalkhoran, Richard Taylor, Mike Drury, Trevor Stain, Bruno Merk

**Affiliations:** 1 School of Engineering, University of Liverpool, Liverpool, United Kingdom; 2 Dalton Nuclear Institute, University of Manchester, Manchester, United Kingdom; 3 Lucid Catalyst and Terra Praxis, Solihull, West Midlands, United Kingdom; 4 School of Physical Science, University of Liverpool, Liverpool, United Kingdom; Universiti Teknologi Malaysia, MALAYSIA

## Abstract

Background: Thurso/Caithness in the United Kingdom has gone through a lot of changes and transitions in the last decades. The decision to build a nuclear reactor test facility in the 1950’s in Dounreay/Caithness UK, as well as the current phase of decommissioning impacted not only the technology development but also the social fabric of the community and individuals within it. This study aimed to explore the lived experiences of people impacted by the nuclear project at Dounreay. The results will form the basis for the discussion about locating future experimental or development facilities, possibly at historic sites. This study employed an exploratory qualitative research approach based on the constructivist grounded theory methodology. Constructivist grounded theory applies a systematic, inductive, iterative, and comparative approach to investigate the meanings behind people’s experiences. It was chosen as the method to explore an under-researched area: the host community for nuclear research in Thurso/Caithness. Purposeful snowball sampling from March 2023 till November 2023 through gatekeepers, media and social media was used. 19 participants including 10 women and 9 men in the age range 36–71 took part. The semi-structured interviews were conducted via phone or online platforms. Participants fondly recalled the hey-day when the Dounreay site was built and the population increased rapidly. They shared memories of how the town of Thurso/Caithness was thriving then which had a huge impact on individuals’ opportunities to receive a very good education, earn a good salary, indirect benefits beyond the nuclear project, and a cosmopolitan sense of community. However, the changes over time and the process of decommissioning had more complex implications for individuals as well as for the community. The work opportunities are still favourable. However, this study also highlights new challenges such as decaying infrastructure, a lack of hope of prosperity, and a feeling of being forgotten. This unique study highlights how a politically driven project impacts a community fundamentally. We identified two themes: mostly positive nostalgic views and Changes and Challenges for the community.

## Introduction

Thurso (town), based on the northern Scottish Highlands coast in the county of Caithness, Scotland, United Kingdom (UK), dates back to at least the 11th century and was a thriving ‘norse’ port town (belonging or relating to Scandinavian countries in medieval times) [1], connected internationally through its fishing and farming industries, and was well-known for linen-cloth and tanning. However, it underwent significant depopulation in the middle of the 19th century and poorhouses were built to host people in need [2]. The trend of population decline continued in the 20th century and Thurso was inhabited by around 3000 citizens in the 1950s [3].

This changed significantly when the Dounreay Experimental Research Establishment (DERE), a nuclear technology development facility, was built in the 1950s [3]. The population of Thurso between 1955–58 “expanded rapidly, from around 2,500 to about 12,000, as the nuclear plant attracted skilled migrants from all parts of the United Kingdom” [4] and beyond [2]. This proved very important for locals looking for new post-WW2 work opportunities [2]. The project attracted new settlers from local, national and international regions, and Thurso began to be referred to as the ‘Atomic Town’ at the time. The technical and career-driven migration (the migrants were colloquially called ‘atomics’) to this unique nuclear project led to a substantial increase in population, not only in Thurso town but also in Caithness county and reversed the depopulation trend of the 19th and first half of the 20th century. Between 1951 and 1961, a population increase from 22,710 to 27,370 (a 17% increase compared to the national trend of 2.5%) was reported from Caithness county [2, 3, 5].

Selecting Dounreay in Caithness County as the project location proved to be a significant advantage for the region. In an area heavily dependent on farming and fishing for employment, the advent of technical modernisation led to a scarcity of jobs. Stephen Cashmore [6] elaborates:

"For a considerable time, Caithness had been losing its most valuable resource—its people. The establishment of Dounreay, however, promised a transformative impact. It not only fulfilled that promise but also repatriated homesick individuals and introduced a new demographic known as the ’Atomics’”

(p. 8).

The construction of housing tailored for atomic workers in Thurso, given its proximity as the nearest town to the facility, located approximately 15km from the site, played a crucial role in the success of the fast breeder reactor program at Dounreay. Without the appeal of contemporary and affordable homes, attracting skilled workers to the northern reaches would have been a formidable challenge, given the prevailing perception of the area as “bleak and remote” [7].

The promise of jobs for the local people and being part of a new technological development fostered an ‘atomic optimism’ [2]. The atomic optimism was not only evident in Thurso/Caithness but also among top-level scientific experts like Alexander Stanculescu. He is a retired physicist who dedicated his career to nuclear technology, and said in a private conversation in December 2023:

*“From the late 1970s on, my generation of young scientists considered nuclear power as the holy grail: (…) that is making nuclear energy practically limitless to fulfil the world’s energy needs*. *Specifically, I was fascinated by the idea of “breeding”, (…)*. *The Dounreay Fast Reactor (DFR) was such a reactor*. *With its white dome and location at the northern tip of Scotland (…), it was an impressive achievement: an experimental nuclear reactor that allowed us to test the operational and safety assumptions that were made during its design*. *(…) It was a fulfilling experience to be part of such an achievement”*.

Alexander Stanculescu (December, 2023)

This atomic optimism was fostered by how the project was communicated within the society. However, a remote risk and low accident record from the UKAEA compared with chemical and engineering was mentioned by Sir Christoph Hinton (managing director of the UK UKAEA’s industrial group) and served as a reassurance of low risk for the nuclear project [2]. Hinton spoke also about the decision to place the nuclear program at Dounreay and said in a public meeting “clearly only the fact that we consider there is a remote risk would cause us to build a factory in a remote area like this’ [8]. A low risk was communicated to the wider public to achieve acceptance for the nuclear project [9]. According to Hogg [9] a historian, the communication related to the nuclear project, was creating “a facade of certainty (which) was necessary to offer legitimacy for and public trust towards nuclear science” [2].

This communication strategy, advertising the positive aspects of the nuclear project and transparently inform about the very remote risk, might explain the positive attitude towards the project in the society. Ross [2] found statements from some people e.g. an employee: “It was employment and that was the bottom line”; nuclear risk was”totally unheard of, (and local people) trusted the UKAEA” and a farmer said “we had no reason to doubt it … there were hundreds of people there including very high-powered scientists who came up from the south and they worked on the site, if they weren’t scared why was I going to be scared?” [2].

It needs to be acknowledged that nuclear technology was in its early stages and many risks for the society and environment were very much unknown. The decision to build the experimental facility was made in 1954, three years before the ‘world’s largest nuclear accident’ of the time happened at Windscale in 1957. Therefore, little was mentioned about any health threats or risks related to nuclear technology in 1954/55.

This new nuclear project brought wealth, life and education opportunities for many and testimonies tell that “Thurso had at one time the best high school education results in the UK” [5]. However, not everyone benefited from the cosmopolitan influence and some were left behind as Mulholland [5] reported of higher rates of teen pregnancies and deprivation among people not involved in the project. This was in contrast to those who had the opportunity to work at Dounreay and received health care, housing and extra pension [5].

The ‘nuclear optimism’ and the potential job options for many were a technical-driven, but is now also perceived as a social experiment by historians [2, 10].

Additionally, Ross [2] mentioned that the UKAEA couldn’t solely depend on housing as a means to attract employees. Based on sociological studies of the Highlands, migration into the area during the late twentieth century was influenced by factors such as employment, housing, personal considerations, and quality of life. This also encompassed access to scenic areas such as the coast, low crime rates, and the perceived safe environment for raising children. [2] emphasised that the quality of life served as a location-specific incentive employed by the UKAEA to attract individuals to work at the Dounreay site.

The aim of the study is to get a deep insight into the lived experience of the people today, their expectations and problems in their daily lives, and to which extent the historical operation as well as the decommissioning of the Dounreay experimental nuclear site effects their todays live and their expectations for the future. The work is a part of the project “Defining a Draft for a Zero Power Reactor Experiment for Molten Salt Reactors” (later called zero power) and contributes to the iMAGINE Project (SIP) [11]. This finding will form the basis for the discussion about locating the envisaged reactor experimental facility for iMAGINE and give guidance on future interactions with potential host communities, their expectations and the needs for a successful siting. In a more general view, the results should support the thinking processes and the planning of any kind of innovative, critical or large-scale infrastructure within a society. Especially for nuclear, the work will support the discussions around the urgently needed future experimental or development facilities, possibly at historic sites.

### Change of operation and its socio-demographic changes and challenges

Between 1955 and 1994 the Dounreay Nuclear Site provided employment for the majority of Thurso’s (and surrounding regions) working age inhabitants; resulting in a thriving Thurso town and county of Caithness, with amenities and opportunities that were expected to endure decades. However, in 1994 Dounreay Nuclear Site ceased its experimental operations and in 2000 the process of decommissioning commenced. The decision was made arguing that nuclear at Dounreay had no ‘economic case’ (Masood, 1998). Other arguments came from environment and safety concerns; “sources in the UK Department of the Environment say that the site’s controversial management and safety record, and the ensuing negative publicity, were also factors in the decision” [12].

Job opportunities have naturally waned, due to the completion of decommissioning projects [13]. However, the completion of decommissioning activities at the Dounreay is uncertain which challenges the planning of the future employability prospects. Dounreay is still the largest employer in Caithness except for health services and education [14]. Dounreay is currently employing about 2000 people including contractors and its supply chain. Data from the Highland area indicates a concerning trend of a declining younger population, likely attributed to young people pursuing education in universities outside the region [15]. Additionally, the type of work at Dounreay has changed with the decommissioning decision and the ONR [16] describes it as “Today, Dounreay is a site of construction, demolition and waste management, all of it designed to leave the site in a safe condition for future generations” [16].

There is little known that the Thurso population, currently 7390 (2023) (Caithness 25,347, 2021), are immediately concerned for their and their children’s future employment opportunities, however, some changes are expected due to the decommissioning completion process [17].

This demographic shift, coupled with an increase in life expectancy for older residents, is altering the overall population structure. The Highland and Islands Enterprise (HIE) area, specifically Caithness and Sutherland, is experiencing a more pronounced impact compared to national trends [15]. According to the Scottish government Wick and Thurso have the “oldest age structures in Scotland” [18].

This shift is contributing to a rising dependency ratio of 69.4 in Caithness (number of people under 16 and 65+ per 100 people of working age)—(WHO, compared to 65.5 regionally (Scotland) and nationally (UK) 56.2 [19]. The dependency ratio is a demographic indicator that measures the ratio of dependents in society to the economically active population. A higher dependency ratio, as seen above, reflects a higher proportion of dependents in the working population. Additional challenges in Scotland such as declining birth rates, reduced migration, and rural depopulation contribute to societal challenges such as increased demand for health and social care services for an ageing population. A 21% population decline is predicted for Caithness by 2041 [20].

**Fig 1:**
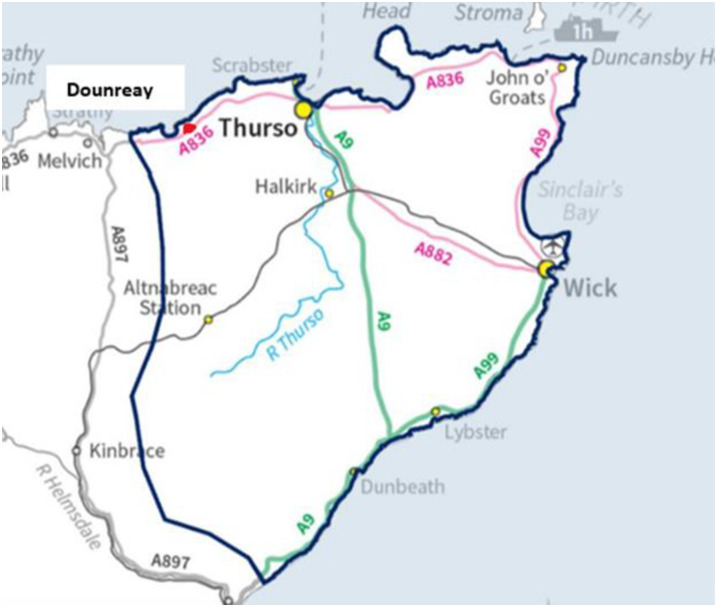
Caithness county. Caithness County Reprinted from [17] under a CC BY license, with permission from [NHS], original copyright [2022]. (Reproduced with permission of NHS Highland, Citation: NHS Highland. Caithness Partnership Profile: Demography and Deprivation. NHS Highland Public Health: 2022).

Another challenge for the county of Caithness (see Fig 1) is deprivation. Caithness has “20% most deprived small areas in Scotland” identified by the Scottish Index of Multiple Deprivation (SIMD). “The SIMD is an area-based measure of relative deprivation rather than household or individual deprivation. The rural deprivation is concerning as SIMD in 2020 mentioned that 11.4% of the population of Caithness were identified as being income deprived and 9.6% of the working-age population were employment deprived” [17].

These demographic and societal changes are intertwined with future career opportunities and lifestyle choices. The ongoing phase of decommissioning, expected redundancies due to decommissioning completion, reduced meaningful job opportunities, and ageing and declining population are potentially impacting individuals, the community and the infrastructure.

In the Zero-power-project, during the identification process of potential sites to host a future experiment, Dounreay was identified as one of the best candidates. To create not only a nuclear science and engineering insight, but also a much wider view, we decided to investigate the following research question: “How did the people living in Thurso/Caithness experience historical, present, and future changes as host community for a nuclear project?”. The core aim was to get a deeper understanding of the interplay of historical political decisions, demographic changes and the impact on individuals and communities.

## Design and methodology

This study employed a qualitative approach to gain a more profound insight into the values, needs, concerns, and experiences of individuals living in Thurso/Caithness area [21, 22]. It is important to undertake “studies” which move away from the top-down approach, favouring elites, towards a historiography incorporating the “local and personal narratives [which] are necessary for an understanding of the British nuclear century” [2, see also 9].

The authors believe that lived experiences are diverse and need to be acknowledged in research related to nuclear [2]. The scarce knowledge of host communities of nuclear projects worldwide led the authors to choose a methodology that is appropriate to identify and co-construct (between researcher and data) new knowledge and theories, constructivist grounded theory [21, see below].

### Principles of constructivist grounded theory methodology

**Fig 2:**
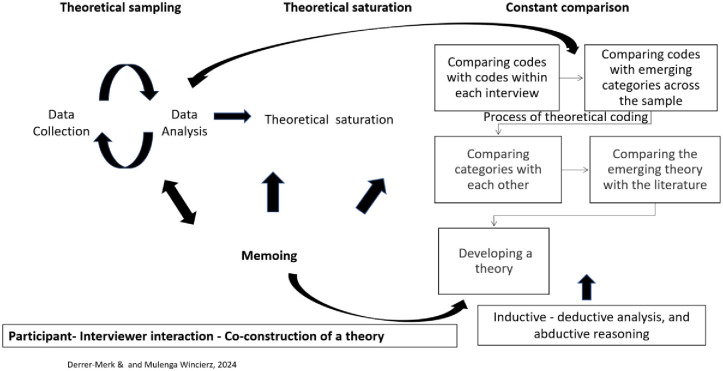
Iterative process of data analysis and theory development in constructivist grounded theory. Iterative process of data analysis and theory development in constructivist grounded theory–the interplay of data collection, analysis, and theory development [28].

**Fig 3:**
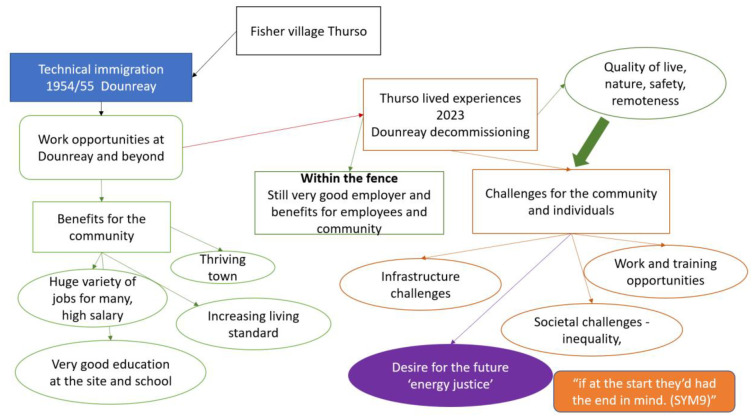
Thurso transition. Thurso transition. The colour reflecting green = positive, red = negative, orange changes and challenges, purple future, and thickness of the arrow = value.

The principles of constructivist grounded theory are important to understand as they guide the systematic research process and differ from other qualitative and quantitative research methods approach with its principles [21]:

- Theoretical Sensitivity refers to the researcher’s pre-existing knowledge and experiences also known as sensitising concepts that guide data analysis without imposing predefined theories and stay in the background till it emerges from the analysis (using preexisting concepts like energy justice in the analysis might risk imposing predetermined findings and constricting the analysis), as Charmaz suggests. [21].
- Theoretical Sampling involves the concurrent collection and analysis of data, where early analysis informs subsequent data collection to refine categories and achieve data saturation.
- The timing of the Literature review suggested by Charmaz [21] supports an initial review to enhance theoretical sensitivity, followed by relevant emergent theoretical concepts during the analysis. Thus, this study was not guided by the concept of energy justice but included it in the discussion as a starting point for new research questions and recommendations for policy and practise.
- Coding is an inductive and iterative process including initial coding to label the data with words or phrases and focused coding to develop overarching categories (Fig 2. Tables 2 and 3)
- Constant Comparative Method ensures rigorous analysis by continually comparing data, codes, and categories to identify patterns and differences across the sample (Fig 2).
- Memoing involves informal notes capturing analytic thoughts throughout the research, aiding reflection, theoretical sampling, and abstraction (refined memos resulted in Fig 3).
- Developing a new knowledge/theory encompasses coding, constant comparison, and the abstraction of categories into overarching themes through imaginative reasoning and the rules of inference (induction, abduction, and deduction) [21].

These principles foster an iterative, flexible, and reflective research process that emphasises the co-construction of how participants and researchers reflect on the given data by asking What does it mean for participants? which supports new knowledge and theory development grounded in the data. More details on how these principles were applied in this study can be found later in the analysis section.

### Quality criteria for constructivist grounded theory (Charmaz, 2014)

This qualitative approach necessitates specific quality criteria distinct from those used in other qualitative or quantitative research. While there is limited consensus on the evaluation of qualitative studies, this research adheres to the criteria set forth by constructivist grounded theory as outlined by Charmaz [21]. These criteria include credibility, originality, resonance, and usefulness.

Credibility is ensured through data saturation, which occurs when additional data no longer yield new insights, thereby sufficiently answering the research question and applying the principles of grounded theory. **Originality** involves offering new perspectives on the phenomena under investigation, particularly important given the limited understanding of host communities in nuclear projects. **Resonance** is achieved when the developed concepts accurately reflect the data, as demonstrated in our findings (see Fig 2). **Usefulnes**s evaluates the practical impact of the study, considering its value to participants and its implications for policy and practice. This is demonstrated through the inclusion of participants’ voices and the formulation of actionable recommendations in the recommendation section.

This study establishes the qualitative criteria and its trustworthiness through theme saturation (sufficient data collection) and the rigorous application of constructivist grounded theory principles, including induction, iterative systematic constant comparison, and abduction (Fig 2). It presents a novel perspective by exploring the lived experiences of a host community, affected by a nuclear project, using a qualitative approach. Focusing on the Thurso/Dounreay community in the UK, this research captures and represents participants’ experiences in depth. The findings are invaluable for informing policy and practice, providing stakeholders, industry, and community with essential insights and fundamental experiences to guide future decision-making and community engagement strategies.

### Recruitment, sample, and data collection

The recruitment aimed to conduct interviews with equally men and women aged 18 and over, living in Thurso and the wider area. The total sample included 19 participants: 10 women and 9 men. Recruitment ceased when no new insights emerged, and theme saturation was reached. The age range of the participants was identified as 39–71. (please see Table 1 Demographics).

**Table 1:** Demographics.

Internal code	Gender male, female	Living alone	Age	Working at Dounreay site–employment status
SDM1	M	no	46	Yes
SMM2	M	no	50–60	Managing role but not at Dounreay
KNM3	M	no	60–65	Yes- now retired
KRM4	M	no	60	No–other occupation
CMM5	M	no	65	No—contractor–now retired
MWM6	M	no	64	Yes—now retired
AGM7	M	no	41	Previous, now oil industry
ACM8	M	no	63	Yes- now retired
SYM9	M	no	56	Previously—now academic
SG F1	F	no	55	Self-employed–now retired,
RJF2	F	no	45	Academic—not living in Thurso but grew up there
RWMF3	F	no	71	Yes- now retired
ISF4	F	yes	58	Self-employed consultant
CFF5	F	no	39	Not working
JCF6	F	unknown	63	Social service
VAF7	F	no	70	Housewife
FYF8	F	no	60	Retired from health care
FBF9	F	no	46	Yes
PMJF10	F	no	53	Health service

Demographics–anonymised participants, gender M = male, F = female, living status, age, Working at Dounreay or elsewhere—employment status

At the start of the recruitment process, we did not know how many participants would volunteer to be interviewed. Therefore, we used a wide range of advertisements and personal contacts to invite participants and were open and flexible to include as many as possible. The initial contact was undertaken through gatekeepers known to the PI and the members of the zero-power team. The first author sent emails and messages with the advert attached asking for sharing the study to recruit participants. A purposeful snowball sampling strategy was used between March 2023 and November 2023, using various methods to reach out to the community of Thurso/Caithness such as media, social media, and presentations at the Dounreay socio-economic stakeholder group.

The interviews were conducted via telephone or online meetings by the first author. Each interview took between 30 and 60 minutes, was audio-recorded and transcribed verbatim externally. The anonymisation was undertaken during the transcription.

The authors were interested in exploring the diverse lived experiences of people in Thurso/Caithness. As we were also interested in why people left the area, it was decided after the first few interviews to also include people who had previously lived in Caithness and wanted to share their experiences. Two volunteers, who did not live at the time of the interview in Thurso/Caithness but either grew up there or had a personal interest to support the study were subsequently interviewed. In compiling the list of participants, the authors recognise that ‘Generating data in qualitative research is about strategically selecting participants whose views and experiences can add meaning to, illuminate, and, in some cases, help explain the phenomenon under study’ [23] (p. 987). This form of ‘open recruitment’ is not bound to previous sample expectations, but rather led by the intention to support the co-construction of new knowledge is possible within the constructivist grounded theory approach and is called theoretical sampling [21].

### New challenge—Imposter participants in online recruitment

This study suffered from the challenge of imposter participants due to online recruitment, thus it is worth drawing attention to this phenomenon. Imposter participants are a relatively new and increasing threat to data integrity in online qualitative research studies [24].

The first imposter participant appeared after the study’s advert went online on LinkedIn. During the interview, suspicions arose as the participant didn’t turn on the camera, lacked knowledge about local specifics, and gave generic answers. Following this incident, the team discussed with the ethics committee and implemented a screening strategy for subsequent interviews, incorporating questions related to the area, health services, travel, and employment opportunities.

In subsequent interviews, participants answered the screening questions. After one participant shared the study on social media, the first author received an influx of ~130 emails within 3–4 days. Some emails lacked formality, had similar wording, or presented fake affiliations (discovered through scrutinizing the given information), and demanded a quick response, none of which were mentioned in the advert. The first author brought this to the notice of the concerned participant who removed the information from their social media account leading to a halt of such emails. After checking each email and discussing the issue a second time with the ethics board the authors decided to include a more intense screening and adapted the inclusion procedure as part of the information sheet and ethics approval, see below:

“Prior to commencement of qualitative research interviews, participants will be asked to show their face and identification. This will not be recorded, and will just be used to verify that the participants are genuine. In order to avoid discriminating against those who do not have photographic identification, any item which contains their name and address (such as a letter, bank statement, will be accepted). Once this step is complete, participants may disable their camera again if they wish. This step is securing that participants meet the eligibility criteria for the study.

(information sheet) “

Participants who did not adhere to the procedure or could not answer the screening questions were excluded from the study.

### Interviews

The interviews were semi-structured, since the aim was to learn what was important to the participants. Before beginning the interview, respondents were sent an information sheet to read, and also asked to sign a consent form; confidentiality and anonymity were additionally assured before commencing the interview. The data was anonymised and each participant was assigned an internal code such as the initials, followed by either F-female or M-male and a number. The approach was, we listen to your experiences and make your voices heard. The study was interested in two broad questions: What is your experience like of living in Thurso/Caithness? and ‘How do/did you feel?’ Supplementary questions were often added such as e.g.: Did you grow up in the area?; How are you connected to the community?; What activities are you involved in?; What are you thinking about the work opportunities?; How has your experience been with the changes over time? (see S1 File).

### Ethics

The ethics was approved by the University of Liverpool No: 12114. The authors did not anticipate being challenged by imposter participants but were closely collaborating with the ethics board of the University of Liverpool to establish appropriate procedures.

### Data analysis

The authors’ philosophical stance is rooted in the belief that lived experiences are multiple, and that these experiences and the attributed meaning are co-constructed by society and individuals, deserving recognition and appreciation. This perspective aligns with Charmaz [25], who accounts for the researcher and the data to study as part of the research process whilst co-constructing new knowledge. To explore the experiences of individuals residing in Thurso and its surroundings and to get a better understanding of the lived experiences, the authors used a qualitative, inductive, and constructivist grounded theory approach by employing iterative constant comparison (see Fig 2) [25, 26]. The approach emphasises that new knowledge is co-created by participants and the researcher without drawing on existing frameworks or theories in order not to constrain the data analysis, thus grounded in the data [25]. In the initial phase, the first author simultaneously listened to interviews while reviewing the anonymised transcripts for accuracy. Subsequently, the interviews were analysed line-by-line to form a comprehensive impression. At this stage each line was labelled with a code (Tables 2 and 3). Simultaneously, the first author created a codebook and memos for each participant, aiding in the analytical process of co-construction of theoretical categories and ensuring reflexivity and analytical rigour, as advocated by Charmaz [21]. This method of analytical line-by-line coding served the incorporation of emerging topics into earlier segments of the interviews. Conducting the interviews and analysing the data was undertaken simultaneously (theoretical sampling), this allowed data-gathering specifically relevant to emerging categories Charmaz [25, 27].

**Table 2:** Mostly positive nostalgic views.

Theoretical constructs of: Mostly positive nostalgic views	Work and training opportunities	Social life and community
Categories	Very good employer, good salary, education, skills beyond Dounreay	New houses, social club, community spirit, very good life
Code examples	Good salary, training, creating work opportunities	Sense of communication, partying, scenery, good lifestyle, impact of Dounreay, social life inequality

From codes to theoretical constructs: Mostly positive nostalgic views—participant’s memories of the hey-days of Thurso/Caithness

**Table 3:** Change and challenges for the community.

Theoretical constructs of: Change and challenges for the community	Quality of life	Employment, Desire for sustainable job opportunities and nuclear projects	Infrastructure challenges	Societal challenges, inequality	Advice and desire for the future
Categories	Sense of community, landscape, nature, family, affordable homes, safe environment	Very good employer, lack of meaningful jobs for the future	Health service, roads, tourism, shops closure, travelling	Forgotten, increased poverty, increased drug addiction	Maintain meaningful jobs, safety
Code examples	Affordable housing, safe place to live, beautiful nature, remote place, sense of community	Impact, divers industry (oil, gas, distillery) “within the fence”	Missed opportunities, health support -service	Forgotten, ignored, unequal treatment, lacking GP and maternity service, poverty, drugs	New use of Dounreay, create new jobs, hope for the future, improve infrastructure

From codes to theoretical constructs: Change and Challenges for the community–Current challenges of lifestyle and future desire

Following the line-by-line coding, focused coding was employed, guided by the process of iterative coding, constant comparison, and being flexible, to identify categories grounded in the data (also known as theoretical coding) (Fig 2, Tables 2 and 3) [21]. Once categories were discerned for each interview, the transcripts were compared to uncover overarching themes and commonalities or differences across the sample. The co-constructed codes, categories, and themes were regularly discussed among the authors. To achieve levels of higher abstraction, abductive reasoning was employed. Abductive reasoning is a form of logical inference that involves generating the best explanation for the studied phenomenon (Tables 2 and 3) [21]. Although the authors were aware of the three-tenet approach of energy justice, encompassing procedure, distribution, and recognition [28, 29], it was used during the analysis merely as a sensitising concept and did not guide the analysis. The concept of energy justice was revisited only because the reflection of what was learned during the study, led to a more community-inclusive discussion and recommendation for the future. Our primary goal was to capture a wide array of experiences from diverse backgrounds and not be constrained to one theoretical concept. For more detail on how the analysis was undertaken please see Fig 2. Iterative process of data analysis and theory development in constructivist grounded theory.

Tables 2 and 3 provide a comprehensive overview of the inductive and iterative coding process, detailing how the analysis in this study was systematically grounded in the data. The tables delineate the analytical steps undertaken, beginning with the initial coding phase, where data segments were meticulously examined and labelled with descriptive codes. This initial phase served as the foundation for identifying overarching categories, which emerged through the grouping of related codes. These categories were then synthesised and refined into broader themes, leading to higher levels of abstraction. Ultimately, these themes were integrated into overarching theoretical constructs that provide a deeper understanding of the studied phenomenon. This structured approach ensured that the theoretical insights were deeply rooted in the empirical data, enhancing the overall quality criteria and particularly the trustworthiness of the research findings. Two higher theoretical constructs emerged from the analysis: **mostly positive nostalgic views** (see Table 2) with the subthemes of work and training opportunities and social life and community; and **challenges and changes for the community** (see Table 3) with subthemes of quality of life, employment and desire for sustainable job opportunities and nuclear projects, infrastructure challenges, and societal challenges–inequality.

## Findings

Many participants talked about their experiences or shared the stories they heard from relatives and friends about the hey-day of Thurso due to the installation of the experimental nuclear site and its implications. Many of them related their experiences to the beginning of the Dounreay project very positively. This included career opportunities, high population influx, and social and infrastructural changes. The overall impression during the experimental phase of the Dounreay site was also very positive and participants spoke about the benefits they gained from the project. However, the recall of the experiences changed when participants shared their views about the decision to decommission the site at the end of the experimental phase. We will provide in the following more details on themes of mostly positive nostalgic views and on the Changes and Challenges for the community.

### Mostly positive nostalgic views

When the decision to build this nuclear experimental reactor was made in the 1950’s, Thurso was chosen to provide housing for the many workers to come including the wider local community [2]. This village was most appropriate as it was close enough to commute every day and far enough to maintain the safety of the inhabitants [7, 18]. Thus, the community in Caithness faced a turn-around as the construction and operation of the Dounreay brought many new working opportunities and related educational opportunities [2]. These opportunities were described very vividly by the participants of this study.

#### Work and training opportunities

LCF6 talked about how the Dounreay site changed her family’s lifestyle for good, acknowledging that both her parents came from a farming background with very few skills but found good paid jobs and careers at Dounreay:

“But when Dounreay came, my mum got that opportunity to start at Dounreay and built up from being a basic secretary to running Sellafield’s student accommodation. And my dad got the opportunity from being a labouring monitor to training in being an instrument technician. So that gave our family a good lifestyle.”

(LCF6)

The working opportunities provided by the Dounreay site did not just offer a job for many but an education starting with a training course followed by studying a degree at the university. Another participant, CMM5, highlighted the fact that the site facilitated an education program for employees with far reaching skills which could be transferred to other industries.

“So all that opportunity came about through going to Dounreay as a 17-year-old, as a scientific trainee. I studied chemistry—I did a degree in chemistry through [Town] College and [University], and all that came about because of Dounreay and was funded by Dounreay.

(CMM5)

Another participant WMM6 talked about of how a friend, his son-in-law, and people beyond the locals benefited from the Dounreay training and said:

“So there are people all over the world that have benefited from Dounreay. (…) You know, it’s not all about just the local, but even one of the top engineers in Formula One did an apprenticeship with the United Kingdom Atomic Energy Authority as well.

(WMM6)”

The apprenticeship was not just any kind of education but experienced by many as a very high level and quality education:

“I would also say that the apprentice training scheme that they had at Dounreay was first class. You know, really first class.

(WMM6)”

As another example of how the training at the site facilitated a participant’s future career and how they could transfer the skills gained at Dounreay, AGM7 said:

“When I left school I didn’t know what I wanted to do so I got on one of these training schemes at Dounreay for one year, and went to the IT section, the health and safety section, the administration section, (…) so that was in 1998 I started there, I just started doing health and safety in 2004, and then in 2012 left Dounreay and went to [city] for a couple of years to work for the oil companies, and then came offshore.”

(AGM7)

Many participants talked about the positive impact of the nuclear site which brought benefits for various generations and the wider community.

“So I have nothing negative to say about Dounreay. We weren’t affected by ill health by it. It brought prosperity to my mum and dad. It brought prosperity to myself and my brother. It gave people in Thurso and North Highland lots of opportunities because there was a lot of income then from Dounreay. So if you weren’t working there you were indirectly supported by it.

(JCF6)

Another participant described Dounreay as the ‘silver lining’ and said:

“Dounreay was in its heydays before I probably even started working there, it was almost, like, seen as the silver lining, it was this place where people could go to work, it paid great salaries and things like that, they had social clubs.”

(FBF9)

The experimental site not only provided lots of well-paid jobs and career opportunities but also invested a lot of money into the local community which another participant, KNM3, said should be recognised despite increasing criticism:

“Dounreay over the years has invested a lot in the community, even during the decommissioning, a lot of money has gone into the local college, a lot of money has gone into the local port to help them develop. So Dounreay has been good for the area, and I wouldn’t like people to forget that.”

(KNM3)

Overall, the training and work opportunities were praised by many participants as this brought a ‘good life’ for many and extended beyond directly working at the site as many skills were highlighted to be transferrable into other industries. Additionally, significant investment has gone into upgrading the region, community building, or just creating business opportunities as a result of people having good money to spend.

#### Social life and community

The decision to build a nuclear research site at Dounreay was technical only. However, it impacted the community in Thurso/Caithness significantly. There were about 1000 new houses built for the ‘atomics’ and the impact on the society and community was explained with a nostalgic perspective by many participants.

One participant who came to start working at Dounreay lived initially in a hostel built for singles and he said:

“The hostel had a bar, for example, and there was snooker and that and there was a social club just over the road from it as well. So yeah, everything was there for us, but we didn’t have much money. And the aim really was to move out because you got all your meals, you know, so you didn’t have to do anything, really. But it was a good time. I would say it was a good time.

(WMM6)”

How lively the community spirit was experienced was told by FBF9 when she recalled her childhood memory.

“I certainly remember when we were children, these social clubs were the hub of lots of family activities. You know, there would be pool halls, there would be a bar area for the adults, they would have where all the kids’ parties and things would be hosted and things like that, Christmas parties, dances.”

(FBF9)

The nuclear research plant at Dounreay was experienced by many participants as beneficial for themselves and for the community. The training and employment opportunities were highly valued and welcomed at the time when the decision to build the test reactor was made. It brought mostly wealth and prosperity to the local community. Some literature suggested that deprivation for those who did not work within the fence of Dounreay (within in the fence means: Dounreay employees and external contractors and the wider supply chain) were disadvantaged. Such kind of inequality was only mentioned by two participants in this study.

“So it was a really bad gender disproportion at [nuclear site]. It was about nine to one male to female. Which had its own problems. So, a lot of the wives, or in some cases the husbands, they might be teachers, or they might work in the banks, or elsewhere, but I think the salaries at [nuclear site] in the ‘80s were significantly higher than what you were going to get in other towns. And I suppose that pushes the level up, but it also creates an inequality.”

(RJF2)

Another participant recalled his school time and spoke about some students’ attitude who came to Thurso. Even he did not speak about the disappointment of being devalued, it can be interpreted between the lines.

“Look if we hadn’t come here, you’d have been forgotten about. So just be grateful to us.” Yeah, I think, going back to my school time, you would sense that from the kids that had come up here from the south, that they were obviously getting a bit of a line from their parents. “These local people should be grateful that we’ve come up here…” yeah. And that wasn’t a predominant theme by any means, but you certainly would come across “

(CMM5).

Many participants talked about their memories of when the Dounreay site was first built and operated in the experimental phase. Most of them remembered it with fondness and positivity. They shared how they, as well as the entire county, gained from the project. In general, only a few participants mentioned any negative effects.

### Changes and challenges for the community

Initially, there were no plans to stop the experiments at Dounreay, however, a political decision was later taken to shut down and decommission the facility.

The announcement came as a disappointment for many but was expected by Britain’s nuclear industry [12]. While the number of employment opportunities was not projected to change significantly as a result of this decision, the type of work has naturally changed as decommissioning primarily involves construction work rather than scientific developments. The changes and challenges shared by participants in this study were not as positive as compared to the memories of the past. As the changes are strongly intertwined with the development, operation, and decommissioning of the nuclear facility (e.g. reduced hope of a prosperous future, reduced transport system due to reduced necessity [see flight times at Wick airport], declining and ageing population fostered economical decision to close health services etc.) This change of operation brought many changes to the community in the following years such as change of employment and reduced training opportunities, infrastructure challenges, societal challenges–Inequality. The quality of life was mentioned by many participants in the study and often it was highlighted as a major aspect to staying in the area. Evidence for each aspect will be presented in the following.

#### Quality of life

Participants often mentioned that different people are coming to the area, some who love the ruralness and others who might not stay long due to the remoteness. One participant especially moved to Thurso three years ago as she wanted to live at such a remote place.

“So, we wanted to be rural, the housing up here is a lot cheaper, you get a lot more space, a lot more house for the money. (…) I’ve become attached to the beaches and cliffs and that kind of thing instead (of hills).

“(PMJF10)

Another participant praised the nature and spirit of the community and stressed that these are reasons for staying in Thurso. But she also acknowledged the challenges due to the remoteness which will be discussed later.

“For me, I mean, I think it’s a great place to live. You know, that’s why I’m still here. (…) It’s a great place to raise a family. It’s a safe environment (…). You know, you have beautiful beaches, you’ve got beautiful greenery, you’ve got coastal walks, you’ve got outdoor water sports. (…) but you have to accept that it is quite remote, but some people like that. A lot of people look for that. And there is a real sense of community as well.”

(FBF9)

Another participant used the term ‘abuse the privilege’ referring to the pleasure she experiences of living in such a remote place and talked about how the place is perfect for her.

“But I abuse the privilege of being here in a remote place. I live half a mile away from anybody. My nearest neighbour is half a mile away, which today, nobody lives half a mile from their nearest neighbour. We live in the middle of a moor in a little old house. It is just perfect.”

(AVF7)

Women and men equally share the joy of living in Thurso/Caithness. One participant who was seconded to work at the Dounreay site for two years recalled his experience with joy and said:

“So yeah, I really enjoyed… when I left the job, I stayed for an extra 6 weeks at my expense because I was enjoying up there. I think if I could have stayed longer, I probably would have.”

(SYM9)

The interplay of safe environment, the secure employment at Dounreay’s research facility, community spirit, but also an overall better and affordable lifestyle was mentioned by many e.g. WMM6 said:

“I still obviously have family down in [central England], but I wouldn’t go back there. I just have a somewhat better lifestyle. It might be remote, but, yeah, it’s a much better lifestyle. (…) I don’t think I could afford the house that I’m in now… (…) I feel safer. (…) I had job security as well for 40 years. And I’ve got friends, etc. When I started at Dounreay, there were eight of us who started at the same time, and there were three of us left out of that eight, and we’re still friends now. So, yeah, I’ve got my friends and family as well that keep me up here. (…), this is far better, you know (…). As I say, it’s got everything that I need.”

(WMM6)

The delight of residing in Thurso/Caithness was passionately conveyed, and numerous individuals couldn’t imagine calling any other place home, even though some participants discussed the inherent challenges.

#### Employment, desire for sustainable job opportunities and nuclear projects

The decommissioning phase started after 2000 and was initially aimed to be finished by mid-2025. However, due to lots of challenges of hazardous material, this has been significantly delayed. An endpoint for at least a brownfield is yet to be named.

*Employment*. There are still opportunities for the foreseeable future to work at the Dounreay site and one participant highlighted the support people can get beyond their role at Dounreay:

“I guess it is keeping certain people employed just now for doing the decommissioning part. But I think Dounreay has got, (…) but some kind of gateway into work, that if you were involved with this, they would help you get work when it is decommissioned.”

(CFF5)

Many participants praised the Dounreay site as a very good employer and education facilitator. This perception was evident in all ages and gender.

“Dounreay, if you can get into Dounreay, is a fantastic employer, and people are on very good salaries and good wages.”

(WMM6)

This view was also supported by another participant, reflecting the wide range of job opportunities provided at Dounreay compared to other industries.

“But I think there’s not many huge businesses that people from all sorts of backgrounds, education, work wise can actually do, whereas Dounreay does seem to have a whole mixture of professionals, people with very specific qualifications or whatever, as well as a lot of people that don’t have the qualifications but are still able to get a job and get a career and work there for a good salary, and good holidays, and good pay and conditions, it’s certainly a big difference.”

(PMJF10)

*Desire for sustainable job opportunities and nuclear projects*. Many participants talked about the desire to establish new nuclear work opportunities, and mentioned the loss of skills, the political opposition to nuclear and the desire from the community to have such an opportunity as RWMF3 and KNM3 said:

“Unless, I mean we’d be desperate to get a new nuclear facility up here but of course the longer it’s left, more and more of these people have retired now. So a lot of the experience that was there is gradually going.”

(RWMF3)

“The community would welcome a new nuclear reactor here for power generation. Good. However, the Scottish government does not support nuclear power. So we have a community, I think, that would welcome that but a political driver that says no.”

(KNM3)

Others desired to have sustainable job opportunities as one participant said:

“So it would be nice to see some sort of industry base that would sustain good jobs in the area rather than rely fully on tourism and farming. (…) Atomic Energy Authority could have done more over time to support the local area by encouraging a bit of diversification from Dounreay. And I don’t think it really happened.”

(CMM5)

Another participant talked about how meaningful job opportunities would help individuals and the community to overcome the increasing poverty and deprivation and compared the current situation with historical decisions and its impact on communities:

“It’s about giving people a reason to get out of their bed in the morning. And if we had an employer come up here, whether it’s another nuclear reactor offering people jobs and skilling them up for decades, and then it’s being passed on to their family, that’s what we need. In the case of Dounreay, and what I see from the economic deprivation, is the same as what happened when we closed the mines, when Maggie Thatcher closed the mines, and you see the deprivation and the lack of hope happening.”

(LCF6)

The employment and training opportunities at the Dounreay site are experienced as very good compared to other industries. However, many desired to host any new nuclear projects and additionally suggested creating more diverse industries to reduce the dependency to one big employer. The quality of work opportunities was mentioned by some with a desire for meaningful job opportunities.

#### Infrastructure challenges

When people were asked how they experienced life at the moment many referred to the lack of health services and health support. This issue seems to be going through all ages and genders. Other aspects such as ‘poor road’ conditions, insufficient transport system, lack of infrastructure for tourism and long drives to Inverness for hospital appointments and shopping were mentioned by many.

*Healthcare*. One participant mentioned the infrastructure difficulty for women in labour. The previous maternity wards in Thurso and Wick closed and women nowadays have to drive to Inverness, which is 120 miles drive from Thurso. RWMF3 said:

“So, if you’re a woman in labour and the snowploughs have been out, you can’t get through. They’ve shut the snow gates. Helicopters can’t fly because of the weather.”

(RWMF3)

The worry for women in labour is expressed often by participants and causes not only a challenge but also a health threat for the woman as well as the baby due to lack of health access.

“But I’ve got a friend at the moment who’s having her first baby and it’s a real worry for them because, (…), you’ve got a partner who’s there to support you and worried about you as well, but having to drive safely to get you to hospital whilst being worried about you. Yeah, it’s a real challenge for us and other people.”

(FBF9)

Another evidence of the difficulty relates to dentist care service and SGF1 talked about the bigger picture of the NHS health service and referred to it as signs of neglect:

“If you see people struggling with dentist appointments and just teeth are rotting away, that’s one of the most visible signs of neglect of healthcare.”

(SGF1)

The challenge to access health services is not only mentioned concerning women in labour and dentist appointments but to other health issues and diagnostic procedures. The mentioned lack of accessible health care service is intertwined with the remoteness, closing of diagnostic wards in Thurso and Wick, costs for transport and accommodation, and challenging transport/road system. One participant relates the challenges with the feeling the county is being ignored and KNM3 said:

“Yeah, so my partner has a hospital appointment in Inverness, so we are going to do 220-mile-round drive to be at the hospital for half past 9 in the morning. So, we have to leave here at about half past 6 in the morning, maybe 6 o’clock, to make that drive. I could catch a train, do the 4 hours on the train, but it will arrive after the appointment. (…) Or I have to go down the day before and try and get accommodation, and accommodation in [Inverness] is more expensive than [Thurso]”

(KNM3).

He continued: So I looked at hotel accommodation, but for me in a Travelodge was £200. So you can’t afford to do that for a hospital appointment. So that infrastructure for us, hospitals, healthcare, in terms of dentistry and all those sorts of social things, we haven’t got or it’s declining rapidly because the county, I feel, is being ignored.

(KNM3)

The challenge to access health care service usually increases in the winter or with bad weather conditions when one participant said: VAF7

“My husband had to go down there [Inverness] for an eye appointment. Women in labour get sent down there. Now, the road is blocked in the snow. Councillors won’t come up in the snow, because it is too dangerous, but pregnant women in labour are expected to travel down there. Old people, travelling down there. People needing cancer treatments, travelling down there. There is just nothing up here.”

(VAF7)

*Tourism challenges and road maintenance*. Many participants spoke with frustration about the missing infrastructure related to tourism and the lack of road and transport maintenance.

“I think it’s very poor actually. (…) A very simple concept (North Coast 500), there’s a road there and we’ll just market it–come and drive this road. Nobody has put the infrastructure in to support that.”

(KNM3)

This experience is shared by WMM6:

“I live on a relatively new estate. The roads are all crumbled. You know, so if we can’t maintain the road infrastructure, what chance have we got with anything else? Look at the airport as well.”

(WMM6)

#### Societal challenges—Inequality

Despite the good working and training opportunities at Dounreay, the beautiful nature and scenery many participants experienced challenges and inequality in their lifestyle.

*Inequality*. Some participants spoke frankly about the feeling of inequality and the feeling of being forgotten and not valued e.g. CFF5 and VAF7 said:

“I think there is an inequality. I think we all feel like, if we are above [Inverness], we are kind of forgotten about. (…) So, it’s just about making things a bit more equal for everyone, I guess. (…) But I think that is the biggest thing. We feel a bit lower down the chain, I think.”

(CFF5)

“Our 30,000 voices count for nothing. I am going to say, people feel as if they are being neglected by the council. (…) They are ignoring us. We are an afterthought, not just from the council, but from government.“

(VAF7)

Some participants recognised the pay discrepancy between people working at Dounreay and its supply chain compared with other industries. KRM4 talked about the challenge of retaining staff beyond the nuclear work options.

“So, understanding salary levels in the nuclear industry locally, so at [Dounreay] site and [Vulcan], they’re very good. But it does create an unbalance in the wider community, because salaries are high, so for conventional industries, construction, tourism and such like, it creates difficulties and challenges in retaining staff. So, quite wide ranges. You’ll find people earning pretty low salaries, too, locally, in comparison!”

(KRM4)

*Societal challenges*. Another societal challenge, deprivation, was mentioned with an increase in poverty, drug, and alcohol abuse in the community. KRM4 spoke about the lack of sports activities in the winter season as one reason for these issues.

“But I think sports provision, when you look at adjoining areas, I think we’re lacking there. It’s basically outdoor sports provision that’s catered for, so once you get into winter, and I think that’s where a lot of our historical problems are in the past, with alcohol and probably drug abuse”.

(KRM4)

The increasing drug problem was mentioned also by SDM1 and he said:

“What we see, there is a growing drug problem up here, though, certainly amongst the young people, which is unfortunate. So, there are definitely drugs about!”

SDM1

The Phase of decommissioning is experienced by some as something negative and related to decreasing opportunities which are not the same compared to the experimental phase. However, JCF6 talked about her experiences of working in the social support service stressing on the issue of deprivation and poverty for people who do not work within the fence of nuclear. JCF6 said:

“I feel that there’s been a lot of negativity because Dounreay has wound down, and there’s not the same opportunity. (…) But because they (pensioners) have their pensions from Dounreay, they manage to live, but they’re still classed as in poverty [by Scottish Multiple Index]. Whereas all the young mums that we work with or the families that are on very low incomes or the people that are in addiction, they are what I would call in real poverty.

(JCF6)

Asked what she thinks causes the social problems such as drugs and others, she related it to the lack of hope for a bright future compared to when Dounreay was built and said:

“For me, from what I’ve seen and the people that I work with, it’s lack of opportunity and lack of hope. There’s not the same work. There’s not the same ability to progress after (education).”

(JCF6)

The changes and challenges for the host community were manifold. Interestingly, many participants praised the quality of life in Thurso/Caithness but spoke frankly of their challenges.

## Discussion

This study aimed to explore the historical, present and future impact of the nuclear industry in the community of Thurso and the surrounding area. This is probably the first study which combined the transition of establishing and operating a nuclear project at Dounreay Thurso/Caithness and its subsequent decommissioning, with a qualitative methodology. The historical and demographic changes of the past decades allow to question how people experienced living in Thurso/Caithness community, which hosted the Dounreay nuclear test program with its civil part, the Dounreay fast reactor test site and well as its non-civil part the naval reactor test establishment. There are various important societal questions related to energy and the concept of energy justice has gained prominence in recent years, aiming to illuminate the effects of energy systems on individuals, the local communities, and the larger societies they are part of [30]. The increased attention to the demand for energy justice has led to new studies looking at the principles of energy justice “(1) Procedural justice–relates to the participation of people in energy-related decision-making processes; (2) Distributional justice–concerns the sharing and distribution of energy system benefits and burdens; (3) Recognition justice–seeks to ensure the acknowledgement of marginalised and/or disadvantaged groups about energy systems” [29–31]. Richard Taylor framed it in a recent interview: “people have got to feel that they’ve been allowed to participate at the level that they wanted to. That the benefits of any projects have been distributed fairly and equitably abound a community, and that their rights and values and heritage as a native community have been recognised within whatever solution has been put through” [32].

We did not test the concept rather being interested in what participants experienced and how this could relate to the three-tenet approach of procedure, distribution, and recognition [29, 30, 32].

Therefore, the concept of energy justice can lead to new insights for future nuclear projects. The aspects of procedure (participation), distribution (benefits and burden), and recognition (public values) might not have been considered in the 50ies in the Dounreay project, as participants of this study often mentioned. However, as many people benefited and were aiming for good jobs and a good life within Thurso/Caithness they did not resent the project. The amenities of newly built houses in combination with the atomic optimism after WW2 gave people a lot of hope for a prosperous future. And this optimism is retained in the collective memories of many of the participants in this study.

The decision to build a nuclear test reactor and an experimental site at Dounreay in 1954 brought fundamental changes to the local economy, to individuals as well as to the community of Thurso in particular. Within three years the town grew from a fishing village to a cosmopolitan ‘atomic town’. People came from different areas of the UK and internationally to participate in the ‘atomic optimism’. For many participants, the living standard increased due to the comparatively high salary and other benefits through the project. Thurso became a thriving town with lots of new amenities, sports clubs, a cinema, and a lively social club for all the UKAEA employees. The educational opportunities changed at all levels and went beyond the high school system. Training and even graduate education were often financed or supported by the Dounreay site. Many participants recalled their experiences very positively and enjoyed talking about them.

The decision to stop the experiments at Dounreay (1994) and the start of the phase of decommissioning (2000) brought significant changes and new challenges for individuals and the community. This decision was politically motivated and the concept which we would call now energy justice did not play a major role as participants often mentioned. Many participants expressed the desire to get new nuclear projects into the site but believed that the local Scottish government and political parties were in opposition to the desires of the community. Despite the currently still ongoing existence of many work opportunities within the Dounreay site, some participants spoke about the challenges they face. The biggest challenge—infrastructure—was mentioned by many. This included a lack of health services, road maintenance, and public transport. Additionally, inequality, loss of hope for the future, increasing poverty, and drug abuse were a concern for some. This study illustrates that some participants were experiencing frustration, particularly when people spoke about inequality and expressed feelings of being forgotten.

Interestingly, for many, the quality of life in Thurso/Caithness still outweighs the current challenges.

### What we can learn from the study

This study is unique as no written evidence about the lived experiences had been found that studied the transition of a town impacted by a novel nuclear project in the UK. The specific context of the nuclear optimism, the remoteness of the test reactor site, the sudden decision to stop the experiments, the subsequent decommissioning and its impact on the community needs to be understood. In general, the study describes the up and down of a large-scale research and experimental site/infrastructure at a remote place and its social implications–it could be any kind of technology which demands large-scale investment and highly qualified employees and thus acts as an over-regional attraction point. This is why we believe, despite the specific context (nuclear, Dounreay), that the experiences in this study can be transferred to other scientific or industrial projects. The Dounreay project took place in a very specific time, the ‘nuclear optimism’. Especially after the Three Mile Island accident, later nuclear projects often suffered from massive local opposition, partly even leading to large-scale projects being abandoned. The findings of this study indicate that the general attitude towards a new nuclear project in the Dounreay region is positive, today. Retrospectively, the people recognize the opportunities the historic project has brought to the region as well as the effect of losing out due to the closing of the experimental site and its decommissioning.

The rising question of the 21 century is how can energy research in nuclear or other industries support a community through transitions caused by these large-scale projects in their build-up, and operation as well as their end. The concept of energy justice might provide a paradigm change and foster new developments on how to make communities not only a place where industry can be established but a genuine partner in decision-making by valuing the needs and concerns of society [29, 30]. The energy justice principles of procedure (participation), distribution (benefits and burden), and recognition (public values) are important to foster a paradigm change from top-down to partnership in decision-making. The principles of energy justice can be addressed e.g. by policymakers, industrial stakeholders and community leaders as suggested below.

Recommendations for Policymakers include integrating the principles of energy justice into the regulatory framework that ensures community participation in decision-making processes. Ensure that the benefits of such large-scale projects are equally experienced in the community and address the burdens with support mechanisms such as education and training opportunities. Furthermore, they should invest in local infrastructure such as health care services and road maintenance to foster well-being and quality of life. Industry Stakeholders should prioritise transparent communication and actively engage with local communities to build trust, address their concerns, and directly support local business development. For community leaders, it is suggested to advocate for their constituents by facilitating dialogues between the community and project developers, to ensure the community’s needs and values are recognised and addressed.

To date, there is little known about the experiences of nuclear host communities, and future research is needed to address this gap to achieve energy justice in addition to affordable, sustainable, and reliable energy for all.

## Conclusion

To conclude, this study is of high importance as there is little known about how a political decision about the establishment of a nuclear project might impact a community. This unique study explored the lived experiences of people residing in a host community of a nuclear project, aiding in the understanding of these specific circumstances and providing guidance for other, future projects. This study brought new insights into how the nuclear project in Dounreay was not only a large-scale technical installation but also had a substantial impact on the local community as a whole. Participants enjoyed talking about the heyday of Thurso/Caithness and the benefits they gained as individuals as well as a community. However, the lived experiences from 2023 differed significantly from the memories as many participants talked about the daily struggle with the infrastructure and the feeling of being forgotten. However, the quality of life still outweighs for many the challenges and was highlighted as ‘home is home’. Even the initial establishment of Dounreay in the 1950ies did most likely not intend to initiate a social experiment, this study demonstrated that the nuclear project at Dounreay cannot be separated from social implications such as economy, infrastructure lifestyle and career opportunities. This study has identified a positive recognition of the potential location of a future reactor test facility in the observed community (e.g. Zero-Power project). We recommend adhering to the principles of energy justice whilst being interested and exploring people’s views, values, and experiences as a basis for decision-making of a site as the next step of the Zero-Power project. This innovative strategy holds the potential to instigate a paradigm shift, incorporating community involvement in decision-making processes to better align with the needs and values of a prospective nuclear host community. These insights should be not only taken seriously for the location of experimental facilities but also for larger-scale investments into energy infrastructure.

Future research could delve into the long-term socio-economic impacts of nuclear projects on host communities, specifically examining cultural and political differences worldwide. The methodologies could draw on qualitative, mixed-methods and quantitative research. Additional research could focus on the comparison of other large-scale energy-related projects to learn from their approach. Additionally, interdisciplinary studies that integrate technical, social, and environmental perspectives would be essential in crafting comprehensive energy policies that promote both technological advancement and social equity.

## Limitation

This study was undertaken with online interviews only. More people could have been reached by having face-to-face interviews locally. However, due to the remote location of this place in the far north of Scotland, this was not possible. The theme saturation was reached after 19 interviews were conducted and analysed. The findings of this study are related to the number of participants and their specific experiences. The authors do not assert that the sample is representative or that the results can be generalised. The authors acknowledge that the recruitment was purposeful, and a different sample might have led to different findings. Additionally, this sample comprised a hard-to-reach community, likely due to the authors unfamiliarity with the community, which may have led to some hesitancy in participation. As noted by one respondent, individuals employed at Dounreay had signed non-disclosure agreements, which might have hindered potentially interested participants from taking part. Furthermore, the interviews were conducted between March 2023 and November 2023, and the analysis was confined to this period, not incorporating any subsequent developments or social challenges. This study did not aim to predict but rather was interested in understanding the lived experience, thus does not claim to be predictive.

## Supporting information

S1 File Semi structured interview schedule. (DOCX)
