# Correction: From fishing village to atomic town and present: A grounded theory study

**DOI:** 10.1371/journal.pone.0339115

**Published:** 2025-12-16

**Authors:** The PLOS One Staff

## Notice of Republication

This article was republished on October 6, 2025, to correct an error in the fifth author’s name. The correct name is: Mike Drury. The correct citation is: Derrer-Merk E, Jain L, Noori-Kalkhoran O, Taylor R, Drury M, Stain T, et al. (2024) From fishing village to atomic town and present: A grounded theory study. PLoS ONE 19(11): e0310144. https://doi.org/10.1371/journal.pone.0310144

The copyright statement for this article has also been updated to include the corrected author’s name.

## Supporting information

S1 File Originally published, uncorrected article. (PDF)

S2 File Republished, corrected article. (PDF)
