# Supplementary material for: From fishing village to atomic town and present: A grounded theory study
Source: PLoS One. 2024 Nov 27;19(11):e0310144. doi: 10.1371/journal.pone.0310144 (PMC11602039; doi:10.1371/journal.pone.0310144)
Supplement: S1 File — (DOCX) [file pone.0310144.s001.docx]

**Semi-structured interview Questions Thurso**

**Biographical life**

Are you from Thurso/local area?

What brought your family to this area?

Did they work in local industries?

**Connections to Thurso and vicinity**

Where have you lived in Thurso/area?

Did you or other family members move to Thurso area?

What has ‘kept’ you in Thurso/area?

Are you planning to change it? If so, why did you/your family leave Thurso/area? Did many of the other residents move to the same areas or different places?

**Working life**

Have you/family worked in any of the industries?

Do you think standard of living was different for the workers here than in other places across Scotland?

How do you think might the future work life look like?

**Domestic Life**

What type of activities were/are available for the residents, pub-culture/local clubs/leisure centre/church related/school/Dounreay led?

What do your children think about their future in Thurso?

**Changes**

How do you feel Thurso and local area has changed? How?

What brought about these changes?

How do you feel about?

What changes would you imagine for the future?

**Dounreay site**

How would you feel about a new use of the Dounreay site?

How do you feel about other new industrial sites in Thurso and Caithness?

What would you desire for the future?

Looking back now, would you say that the creation and building the Dounreay site was a good thing or a bad thing or neither, using a minus 5 to plus 5 scale, where minus five means it was a very bad thing, 0 means neither and plus five means it was a very good thing?

How certain are you about this, where 0 means not certain at all and 100 means completely certain?

Would you say that the demolishing of the Dounreay site was a good thing or a bad thing or neither, using a minus 5 to plus 5 scale, where minus five means it was a very bad thing, 0 means neither and plus five means it was a very good thing?

How certain are you about this, where 0 means not certain at all and 100 means completely certain?

**Support**

What could the local community do for you to support you?

What could the central government do for you to support you?

**Advice**

What advice would you give to people in living in the same area?
